# Outcomes of HPV type-specific serostatus do not associate with oral or genital HPV-carriage in non-vaccinated women followed for three years

**DOI:** 10.1186/s12905-022-01717-x

**Published:** 2022-04-28

**Authors:** Salla Vuorinen, Kari Syrjänen, Tim Waterboer, Seija Grénman, Stina Syrjänen, Karolina Louvanto

**Affiliations:** 1 Department of Obstetrics and Gynecology, Turku University Hospital, University of Turku, Turku, Finland; 2 Department of Clinical Research, Biohit Oyj, Helsinki, Finland; 3 German Cancer Research Center (DKFZ), Heidelberg, Germany; 4 Department of Oral Pathology, Institute of Dentistry, Faculty of Medicine, University of Turku, Turku, Finland; 5 Department of Obstetrics and Gynecology, Faculty of Medicine and Health Technology, Tampere University, Tampere, Finland; 6 Department of Obstetrics and Gynecology, Tampere University Hospital, Tampere, Finland

**Keywords:** HPV, Serology, Genital, Oral, Women, Antibody, Persistence

## Abstract

**Background:**

The role of human papillomavirus (HPV) antibodies acquired through natural infection and their role in protection for subsequent cervical or oral HPV-carriage remains unclear.

**Methods:**

A total of 267 women, with a 36-months follow-up, from the Finnish Family HPV (FFHPV) study were evaluated to shed more light on persistent HPV-specific antibodies to genital or oral HPV-carriage, clearance or persistence during the three years follow-up. The type-specific seroprevalence for HPV genotypes 6, 11, 16, 18 and 45 in these women was assessed in relation to the detection of the same genotype or any HPV in their oral and genital samples. The following HPV serological outcomes where detected: being always seronegative, seroconversion or persistent seropositivity.

**Results:**

Genital HPV16 infections were most prevalent at the end of the follow-up (24- and 36-month visit) among women who tested always seronegative for HPV16. No such associations between serology and HPV detection were established for the other HPV genotypes in the genital or oral samples. The development of long-term type-specific HPV 6,11,16,18 and 45 persistence (≥ 24 months) or clearance of the genital or oral infections was not different among the women with high HPV genotype specific antibody levels and those testing always HPV-seronegative.

**Conclusion:**

No significant role was disclosed for the acquired natural high-level- or persistent HPV antibodies as determinants of the genital or oral HPV infection outcomes in these young, non-vaccinated women.

## Background

Most mucosal human papillomavirus (HPV) -infections are transient and clear spontaneously by active immunological response within a few years [1–3]. Still approximately 10–20% of women fail to clear their HPV infection and are at higher risk for progression to pre-cancerous lesions. A persistent genital infection is known to be a key event in cervical carcinogenesis [4]. HPV infections are closely linked not only with the development of cervical cancer but also to oropharyngeal cancer.

HPV infections in both oral and genital mucosa are known to elicit an immunological response producing HPV-type-specific antibodies [5, 6]. However, only around 50–70% of these women do develop detectable levels of HPV- antibodies after a natural infection [7–9]. A recent meta-analysis concluded that HPV antibodies acquired through natural infection can provide a modest protection against subsequent cervical HPV infections among non-vaccinated women [3]. Not unexpectedly, these natural antibody levels are also considerably lower as compared to those are after HPV vaccination [10]. According to a mathematical modelling of a high-risk population, a wide individual variation exists in the duration of HPV infection and acquired immunity, but an acquired natural immunity following HPV clearance might provide some protection against new HPV infections [11]. However, it is still unclear what is the minimum antibody level needed for protection or whether only extremely high levels of acquired natural antibodies can provide any such protection against the future HPV infections.

In this study, we investigated type-specific HPV6, 11, 16, 18 and 45 antibody levels and their serological outcomes (i) persistent seropositivity, ii) seroconversion, and iii) always seronegative) during a 36-month follow-up, with special reference to the different outcomes of genital and oral HPV infections among young non-vaccinated women in a long-term cohort study.

## Materials and methods

### Subjects

The Finnish Family HPV (FFHPV) study is a prospective cohort study conducted at the Department of Obstetrics and Gynecology, Turku University Hospital, University of Turku and at the Institute of Dentistry, Faculty of Medicine, University of Turku, Turku, Finland. The cohort included 329 pregnant women who were recruited between 1998 and 2001 (minimum of 36 weeks of pregnancy) and were followed-up for six years after the delivery. All participants were Caucasian origin and had the same ethnic background. The HPV results of the whole FFHPV study cohort (331 mothers and 131 fathers) have been reported in a series of previous publications, only few cited here [1, 12, 13]. A written informed consent was obtained from all the study participants. The Research Ethics Committee of Turku University and Turku University Hospital has approved the study protocol and its amendment (#2/1998 and #2/2006).

The present study focuses on naturally acquired HPV antibody levels for HPV genotypes 6, 11, 16, 18 and 45 and their association to the genital and oral HPV infection outcomes among 267 unvaccinated women from the FFHPV study [14]. Of the 329 women originally enrolled, 267 women were eligible for the present study having at least two serum samples taken during the study period.

### Samples

Genital and oral scrapings from the women were collected for HPV-testing with a cytobrush (MedScand, Malmö, Sweden) as described before [12]. HPV genotyping was done between the years of 2005 to 2010 by Luminex-based Multimetrix kit (Progen Biotechnik GmbH, Heidelberg, Germany), which detects 24 low-risk (LR)- and high-risk (HR)-HPV genotypes as followed: LR-HPV: 6, 11, 42, 43, 44, and HR-HPV: 16, 18, 26, 31, 33, 35, 39, 45, 51, 52, 53, 56, 58, 59, 66, 68, 70, 73, 82 [15].

### Serology

Blood samples were taken at baseline and at 12-, 24- and 36 months of the follow-up. The samples were collected between the years of 1998 to 2003. All samples were stored after collection first at − 20 °C for no longer than one week, and then at − 70 °C until the analyzed between the year 2008 to 2009 as previously described [1]. Major capsid protein L1 antibodies for HPV types 6, 11, 16, 18 and 45 were analyzed by multiplex HPV serology based on glutathione S-transferase fusion-protein capture on fluorescent beads (also referred as GST-L1 assay), as described previously [16, 17]. This method is frequently used and validated in seroepidemiological studies concerning HPV [18]. Sera were scored as positive when the antigen-specific median fluorescence intensity (MFI) values exceeded the cut-off level of 200 MFI for the L1 antigen of individual HPV genotypes [19].

### Statistical analyses

Frequency tables were analyzed using the χ2 test or the Fisher’s exact test for categorical variables. Differences in the means of continuous variables (i.e. log-transformed HPV antibody titres) were analyzed using ANOVA (analysis of variance) after controlling for their normal distribution [14]. Women’s genital and oral HPV 6, 11, 16, 18 and 45 genotype-specific and any-HPV prevalence at each follow-up visit was compared with the women’s serological status for these same HPV genotypes [14]. Serological status was classified into three categories as follows: 1) Always seronegative (MFI remains < 200 at each visit); 2) Seroconversion, defined by two conditions: (i) an MFI value < 200 in the first and > 200 in the subsequent sample, and (ii) at least a two-fold increase of the previous serum value in any subsequent sample; and lastly 3) Persistent seropositivity (MFI constantly > 200 in all follow-up visits) [14]. The persistence and clearance of oral and genital HPV 6, 11, 16, 18 and 45 infections were compared between two groups of women: 1) those with constantly high-titer (> 400MFI) of HPV antibodies, and 2) women who tested constantly HPV-seronegative. Logistic regression with its Odd Ratio (OR) was calculated as a likelihood of the above serostatus to predict HPV persistence or clearance. Persistent HPV infection was defined as being positive for type-specific 6,11,16,18 or 45 HPV genotype for 24 months or longer during the follow-up. All statistical analyses were performed using SPSS (IBM, NY, USA, PASW Statistics version 26.0.1.) and STATA/SE 16.1 (Stata Corp., College Station, TX, USA) software packages. All statistical tests performed were two-sided and declared significant at the *P*-value < 0.05 level.

## Results

The mean age of the women at the time of enrolment to the FFHPV cohort was 25.5 years (range 18–38). The key characteristics and the dynamics of seroprevalence, seroconversion and antibody decay of these women have been described in detail previously [1, 13].

First, we looked at genotype-specific serological outcomes as related to any-HPV genotype detection in the genital samples at the five follow-up visits as described in Table 1. At baseline, genital any-HPV prevalence was significantly associated with HPV16 always seronegative and HPV16 persistent seropositivity serological status. At baseline, 134 (69.3%) of the women testing HPV DNA negative for any HPV-genotype remained HPV16-seronegative during the follow-up compared to those 47 (24.4%) women who were persistent HPV16 seropositive during the three-year follow-up. No such significant associations were recorded for HPV16 serology at any other follow-up visits, or for the other HPV (6, 11, 18 and 45) serology outcomes. Secondly, we analyzed serological association with the five individual HPV genotype genital infections. Genital HPV16 genotype carriage was significantly more prevalent in women who were always seronegative (women n range 41–52) than in those who were recorded persistent HPV16 seropositive (women n range 11–16). No such differences were established in the other genotype-specific analysis of HPV 6, 11, 18 or 45 (data not shown).

**Table 1:** Serological HPV 6,11,16,18 and 45 genotype-specific outcomes* as related to Any-HPV detection in the genital samples at the follow-up visits

		Baseline		2 months		12 months		24 months		36 months	
HPV type	Serological outcome	HPV + n (%)	HPV − n (%)	HPV + n (%)	HPV − n (%)	HPV + n (%)	HPV − n (%)	HPV + n (%)	HPV − n (%)	HPV + n (%)	HPV − n (%)
	Always Seronegative	10 (24.4)	58 (31.0)	15 (38.5)	53 (28.3)	26 (23.2)	41 (36.3)	40 (32.3)	26 (30.6)	33 (29.2)	30 (34.1)
HPV 6	Seroconversion	2 (4.9)	3 (1.6)	0 (0.0)	5 (2.7)	3 (2.7)	2 (1.8)	4 (3.2)	0 (0.0)	2 (1.8)	1 (1.1)
	Persistent Seropositivity	29 (70.7)	126 (67.4)	24 (58.5)	129 (69.0)	83 (74.1)	70 (61.9)	80 (64.5)	59 (69.4)	78 (69.0)	57 (64.8)
	Always Seronegative	27 (77.1)	160 (85.1)	28 (87.5)	157 (80.1)	80 (80.0)	103 (81.7)	100 (84.7)	78 (83.0)	98 (84.5)	75 (83.3)
HPV11	Seroconversion	0 (0.0)	4 (2.1)	0 (0.0)	4 (2.0)	0 (0.0)	4 (3.2)	1 (0.8)	0 (0.0)	1 (0.9)	1 (1.1)
	Persistent Seropositivity	8 (22.8)	31 (15.9)	4 (12.5)	35 (17.9)	20 (20.0)	19 (15.1)	17 (14.4)	16 (17.0)	17 (14.7)	14 (15.6)
	Always Seronegative	**15 (44.1)**	**134 (69.4)**	20 (66.7)	128 (65.6)	61 (57.5)	84 (71.8)	76 (63.9)	65 (73.0)	74 (65.5)	63 (70.8)
HPV16	Seroconversion	**0 (0.0)**	**12 (6.2)**	0 (0.0)	11 (5.6)	7 (6.6)	5 (4.3)	6 (5.0)	3 (3.4)	8 (7.1)	2 (2.2)
	Persistent Seropositivity	**19 (55.9)**	**47 (24.4)**	10 (33.3)	56 (28.7)	38 (35.8)	28 (23.9)	37 (31.1)	21 (23.6)	31 (27.4)	24 (27.0)
	Always Seronegative	26 (63.4)	164 (77.7)	27 (69.2)	162 (76.8)	85 (74.6)	101 (75.4)	101 (76.5)	77 (76.2)	100 (79.4)	72 (72.0)
HPV18	Seroconversion	2 (4.9)	9 (4.3)	2 (5.1)	9 (4.3)	5 (4.4)	6 (4.5)	8 (6.1)	3 (3.0)	4 (3.2)	7 (7.0)
	Persistent Seropositivity	13 (31.7)	38 (18.0)	10 (25.6)	40 (19.0)	24 (21.1)	27 (20.1)	23 (17.4)	21 (20.8)	22 (17.4)	21 (21.0)
	Always Seronegative	37 (90.2)	206 (91.2)	34 (89.5)	207 (91.2)	112 (88.2)	127 (93.4)	133 (89.9)	95 (95.0)	127 (92.0)	94 (92.2)
HPV45	Seroconversion	0 (0.0)	4 (1.8)	0 (0.0)	4 (1.8)	3 (2.4)	1 (0.7)	1 (0.7)	2 (2.0)	2 (1.4)	1 (1.0)
	Persistent Seropositivity	4 (9.8)	16 (7.1)	4 (10.5)	16 (7.0)	12 (9.4)	8 (5.9)	14 (9.5)	3 (3.0)	9 (6.5)	7 (6.9)

*Serological status was categorized into three groups as followed: 1) Always seronegative, MFI value < 200 at each visit; 2) Seroconversion, defined by two conditions: (i) an MFI value < 200 in the first and > 200 in any subsequent sample, and (ii) at least a two-fold increase of the previous serum value; and 3) Persistent seropositivity, with MFI value > 200 in all follow-up samples. Significant results are bolded

HPV-genotype-specific serological outcomes as related to the any-HPV detection in the oral samples at the follow-up visits are shown in Table 2. The only statistically significant result was found among the HPV11 serological outcome categories at the 2-month follow-up visit; 83.0% (n = 39) of HPV-DNA positive (any HPV-genotype) women were always HPV11-seronegative as compared to 6.4% (n = 3) of those who seroconverted for HPV11. Of the women testing HPV DNA negative for any HPV-genotype, 81.6% (n = 146) remained always HPV11 seronegative and 17.9% (n = 32) showed persistent seropositivity as compared to only 0.6% (n = 1) of those who showed HPV11 seroconversion. The genotype-specific HPV analysis for 6, 11, 16, 18 or 45 oral infections did not show any significant association with the respective genotype-specific serology (data not shown).

**Table 2:** Serological HPV 6,11,16,18 and 45 genotype-specific outcomes* and Any-HPV detection in the oral samples at the follow-up visits

		Baseline		2 months		6 months		12 months		24 months		36 months	
HPV type	Serological outcome	HPV + n (%)	HPV − n (%)	HPV + n (%)	HPV − n (%)	HPV + n (%)	HPV − n (%)	HPV + n (%)	HPV − n (%)	HPV + n (%)	HPV − n (%)	HPV + n (%)	HPV − n (%)
	Always Seronegative	12(32.4)	56 (29.6)	17(34.7)	51 (29.5)	16(30.2)	51 (30.2)	12(27.3)	56 (30.6)	14(27.5)	51 (32.7)	10(31.3)	53 (31.0)
HPV6	Seroconversion	1 (2.7)	4 (2.1)	0 (0.0)	5 (2.9)	1 (1.9)	4 (2.4)	1 (2.3)	4 (2.2)	1 (2.0)	3 (1.9)	0 (0.0)	3 (1.8)
	Persistent Seropositivity	24(64.9)	129 (68.3)	32 (65.3)	117 (67.6)	36 (67.9)	114 (67.5)	31 (70.5)	123 (67.2)	36 (70.6)	102 (65.4)	22 (68.8)	115 (67.3)
	Always Seronegative	28 (80.0)	158 (81.4)	**39(83.0)**	**146 (81.6)**	46 (85.2)	137 (80.1)	33 (80.5)	153 (81.4)	42 (89.4)	137 (83.0)	26 (81.3)	148 (84.1)
HPV11	Seroconversion	2 (5.7)	2 (1.0)	**3 (6.4)**	**1 (0.6)**	1 (1.9)	3 (1.8)	1 (2.4)	3 (1.6)	1 (2.1)	0 (0.0)	0 (0.0)	2 (1.1)
	Persistent Seropositivity	5 (14.3)	34 (17.5)	**5 (10.6)**	**32(17.9)**	7 (13.0)	31 (18.1)	7 (17.1)	32 (17.0)	4 (8.5)	28 (17.0)	6 (18.8)	26 (14.8)
	Always Seronegative	26 (70.3)	122 (64.6)	30 (62.5)	116 (66.7)	28 (62.2)	117 (66.1)	24 (58.5)	124 (68.9)	26 (59.1)	115 (70.1)	19 (59.4)	118 (69.0)
HPV16	Seroconversion	1 (2.7)	11 (5.8)	2 (4.2)	9 (5.2)	2 (4.4)	10 (5.6)	2 (4.9)	10 (5.6)	3 (6.8)	6 (3.7)	1 (3.1)	9 (5.3)
	Persistent Seropositivity	10 (27.0)	56 (29.6)	16 (33.3)	49 (28.2)	15 (33.3)	50 (28.2)	15 (36.6)	51 (28.3)	15 (34.1)	43 (26.2)	12 (37.5)	44 (25.7)
	Always Seronegative	33 (78.6)	155 (74.5)	42 (79.2)	144 (75.0)	46 (79.3)	140 (74.5)	33 (80.5)	156 (74.3)	41 (75.9)	137 (77.0)	29 (80.6)	144 (75.0)
HPV18	Seroconversion	3 (7.1)	8 (3.8)	2 (3.8)	8 (4.2)	1 (1.7)	10 (5.3)	0 (0.0)	11 (5.2)	3 (5.6)	8 (4.5)	0 (0.0)	11 (5.7)
	Persistent Seropositivity	6 (14.3)	45 (21.6)	9 (17.0)	4 (20.8)	11 (19.0)	38 (20.2)	8 (19.5)	43 (20.5)	10 (18.5)	33 (18.5)	7 (19.4)	37 (19.3)
	Always Seronegative	42 (93.3)	199 (90.5)	53 (93.0)	184 (90.6)	58 (89.2)	181 (91.9)	47 (92.2)	195 (90.7)	55 (93.2)	173 (92.0)	35 (89.7)	187 (92.1)
HPV45	Seroconversion	1 (2.2)	3 (1.4)	1 (1.8)	3 (1.5)	2 (3.1)	2 (1.0)	2 (3.9)	2 (0.9)	2 (3.4)	1 (0.5)	1 (2.6)	2 (1.0)
	Persistent Seropositivity	2 (4.4)	18 (8.2)	3 (5.3)	16 (7.9)	5 (7.7)	14 (7.1)	2 (3.9)	18 (8.4)	2 (3.4)	14 (7.4)	3 (7.7)	14 (6.9)

*Serological status was categorized into three groups as followed: 1) Always seronegative, MFI value stayed < 200 with each visit; 2) Seroconversion, defined by two conditions: (i) an MFI value < 200 in the first and > 200 in the second sample, and (ii) at least a twofold increase of the previous serum value; and lastly 3) Persistent seropositivity, MFI value stayed > 200 in all follow-up visits. Significant results are bolded

Genotype-specific serological outcomes (high-titre antibodies (> 400MFI) vs. always seronegative) were compared among women with i) persistent (> 24-month) HPV, and ii) HPV clearance, in the oral and genital samples, separately (Table 3). Because of the small size of the subgroups, ORs were not always calculable. Even when calculable, we could not disclose any significant associations for these serological outcomes to predict genital or oral HPV persistence or clearance.

**Table 3:** HPV genotype 6,11,16,18,45—specific serology (MFI > 400) in women predicting the persistence or clearance of oral or genital HPV infection with the concordant HPV genotype

HPV genotype	Persistence ≥ 24 months (n)		Clearance (n)		OR* 95%CI
	**Genotype specific serology	Always seronegative	**Genotype specific serology	Always seronegative	
*Genital mucosa*					
6	1	1	4	1	0.25 (0.002–39.09)
11	0	0	0	1	NC
16	8	42	4	35	1.66 (0.40–8.16)
18	0	3	1	4	NC
45	0	3	0	5	NC
*Oral mucosa*					
6	0	0	1	1	NC
11	0	0	0	1	NC
16	8	26	6	26	1.33 (0.34–5.35)
18	1	1	0	3	NC
45	0	0	0	0	NC

*NC* OR non-calculable

*OR calculated as likelihood of HPV genotype specific serology positive high-level serology titers to predict HPV persistence

**The 400MFI cut-off

## Discussion

The role of naturally acquired high-level HPV antibodies and their role in providing protections against new HPV-infections is conflicting [11]. Our aim was to shed more light on this issue by evaluating the HPV 6, 11, 16, 18 and 45 antibody levels and their serological outcomes to the clinical outcomes of the genital and oral HPV infections among women during a long-term prospective follow-up.

In these analyses (Tables 1 and 2), surprisingly few significant associations were disclosed. As to the genital site, the status of being always HPV16 seronegative was associated with an increased likelihood of testing HPV16 DNA positive during the subsequent follow-up visits (at 24 and 36-months) as compared with the women who were persistently HPV16-seropositive. This is in alignment with a previous study, where young women with genital HPV16-infection were shown to be HPV16-seronegative [20]. In our study, these genital HPV16 carriage cases were still HPV16 seronegative at the end of the follow-up period, so with a longer follow-up, some of these women might have been seroconverted. Our results are in line with a previous study, which showed that GST-L1 seropositivity did not indicate protection from incident infection over four years of follow-up (HPV16 adjusted OR of 1.72 (95%CI 0.95–3.13) [18]. There is however also evidence that some HPV infections do not always cause seroconversion and even the women with persistent HPV infection fail to seroconvert [7]. Our previous analysis on this cohort showed that half (67/134) of the women with established HPV seroconversion was only with one of the HPV genotype (6, 11, 16, 18 or 45) while the other half had the seroconversion recorded with multiple HPV genotypes [1]. Here we analysed the HPV genotype specific serology stratified to three different categories according to the 3-year follow-up data: always seronegative, always seropositive, and seroconverted. The association of this serology data with the presence of any-HPV or HPV specific genotypes of 6,11,16,18 and 45 carriages in the genital or oral mucosa by visits were analyzed. The present study did not disclose any significant associations between the HPV 6,11,18 or 45 genotype-specific serological outcomes and the clinical course of genital or oral HPV infections.

We also assessed the effect of higher-level (> 400 MFI) HPV 6, 11, 16, 18 and 45 L1-antibodies as predictors of long-term persistence or HPV-clearance of genital and oral infections, using the HPV6,11,16,18 and 45-negative serostatus as the reference. Thus, four subgroups arise: 1) high-titer HPV-persistent; 2) high-titer HPV cleared; 3) seronegative HPV-persistent, and 4) seronegative HPV cleared (Table 3). Is has been hypothesized that high levels of natural HPV antibodies would protect against the acquisition of subsequent HPV infections, although the role of the natural antibodies still remains undetermined [3, 21]. The problem with most previous natural antibodies studies have been the relatively small populations with lack of longitudinal follow-up, different assays, and analytic techniques that may affect the discordant results [3, 18]. In addition, the comparison between different assays is problematic as they do not measure equivalent aspects of the immune response and their seropositivity cut-offs are not calibrated against each other [3, 18]. With our small number of cases stratified into the four subgroups, the ORs were not always calculable for all the five HPV genotypes (Table 3). In fact, OR was only available for HPV16 in both oral and genital site, and for HPV6 in the genital site. For both genotypes the OR values were not significant, implicating that genital or oral HPV16 (and HPV6) persistence or clearance could not be predicted by the respective serological status (high-titre antibodies versus seronegative status). In one previous study, high-level HPV16- or HPV18 antibodies following a natural infection were associated with a reduced risk of subsequent HPV16 and HPV18 infections [2]. Interestingly, the likelihood for conveying a protective effect against the future infection was higher for high-titer HPV18 antibodies than for those of HPV16 [2]. Unfortunately, we were unable to elaborate such data for HPV18, because of the small number of HPV18-infected women. In our previous analysis of these women, we observed that those who cleared their cervical HPV16 infection had the highest titres of HPV16 antibodies, whereas those who acquired incident HPV16 infections had the lowest antibody levels [13]. Somewhat unexpectedly, the long-term HPV persistence in the genital or oral site was not predicted by the negative serostatus in the present study. One must point out that with our small number of women in this study, all women were also pregnant at the baseline visit (at their third trimester). Pregnancy might alter the HPV related serological response but also the viral dynamics, per se. However, knowledge on pregnancy and serological HPV infection response is still scanty.

## Conclusions

With this present study, we were unable to demonstrate that categorization of HPV serology based on three-year follow-up or even using higher cut-off for HPV seropositivity for HPV6, 11, 16, 18 or 45 L1 proteins could confer a measurable protection against the infections by the respective HPV genotypes in young, non-vaccinated women. Some protection by the naturally acquired HPV L1-antibodies is likely, given that the strong antibody responses to prophylactic HPV vaccines are believed to be accountable for the protection against the future HPV infections among vaccinated women [9]. The protective effect of natural immunity is though considered to be inferior to the immunity acquired by HPV vaccinations in protecting against HPV reinfection [3]. Even if this protective effect is likely to be weaker, it will be essential for the future to establish whether these naturally acquired antibodies could also provide some protection and to what extent against HPV-infection persistence and HPV- infection related carcinogenesis.

## Data Availability

The datasets used and analyzed during the current study are available from the corresponding author on reasonable request.

## Abbreviations

HPV: Human papillomavirus
FFHPV-study: Finnish Family HPV study
LR: Low-risk
HR: High-risk
MFI: Median fluorescence intensity
OR: Odd ratio
